# Vulcanized Rubber for Capping Exposed Nerves

**Published:** 1868-01

**Authors:** 


*Vulcanized Rubber for Capping Exposed Nerves.*—Dr. J. R. Walker of New Orleans, in an article published in the November No. of the *Dental Register*, recommends vulcanized rubber caps over exposed nerves, and describes his method of applying them in the following case:

"Having about two years since an unusually difficult case, in which the patient (a lady) was very desirous of having a pulp saved, and being satisfied that nothing which had been been tried, would prove equal to the occasion, it occurred to me that *vulcanized rubber*, ought to answer the conditions, better than anything that had been used.

The tooth was the right superior central incisor, cavity very large, in the labial surface, exposure large, but pulp healthy, and the patient ditto, temperament nervous sanguine. I took a piece of rubber plate, with one side finely polished, and after filing it to the proper thickness, cut out the cap in form to suit the cavity, then, with a fine file, dressed the edges thin, leaving it thick enough in the central portions, to sustain the filling. Having the cavity well prepared, I placed the cap over the exposed pulp, and filled with gold.

Notwithstanding the size of the cavity, and its peculiarly exposed position to all thermal changes, the rubber being a non-conductor, and the thin and pliant edges giving it a thorough adaptation, proved so good a protection, that the patient has never felt any inconvenience, even in drinking ice water. Thus encouraged. I have tried it in, perhaps, fifty cases since, being careful that the pulp shall be always healthy, and although I have used it in each case, with the understanding that it was an experiment, the patient promising to inform me of any want of success, I have not heard of a single failure.

As my experience in this method of saving pulps has been so exceedingly satisfactory, I offer the idea to the profession, hoping that by so doing I may enable others to *save more teeth alive*, and induce them to abandon the too common practice of murdering every dental pulp that has a slight exposure."

